# Supplementary figures and images for: Computer-guided binding mode identification and affinity improvement of an LRR protein binder without structure determination
Source: PLoS Comput Biol. 2020 Aug 31;16(8):e1008150. doi: 10.1371/journal.pcbi.1008150 (PMC7485979; doi:10.1371/journal.pcbi.1008150)

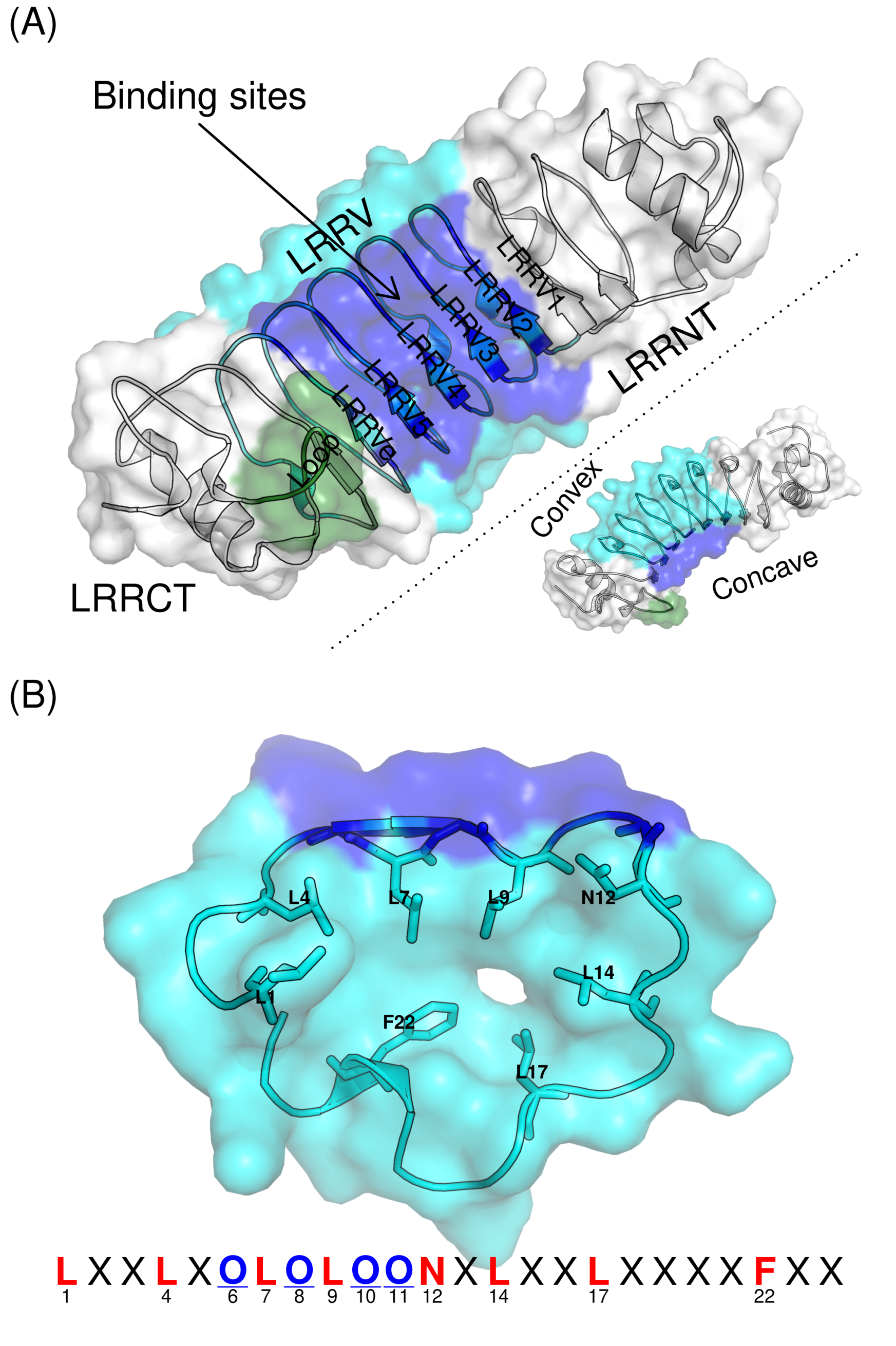

Supplement: S1 Fig — (A) A repebody (Rb) largely consists of three parts: N-termianl cap (LRRNT), variable regions (LRRV) and C-terminal cap (LRRCT). Binding occurs at the concave region of LRRV (in darker blue). (B) Structure of a single LRRV motif, with side chains of conserved residues rendered as stick figures. Each LRR is composed of six conserved leucine residues, a central conserved asparagine residue, and conserved phenylalanine residue on the C-terminal side. (TIF) [file pcbi.1008150.s001.tif]

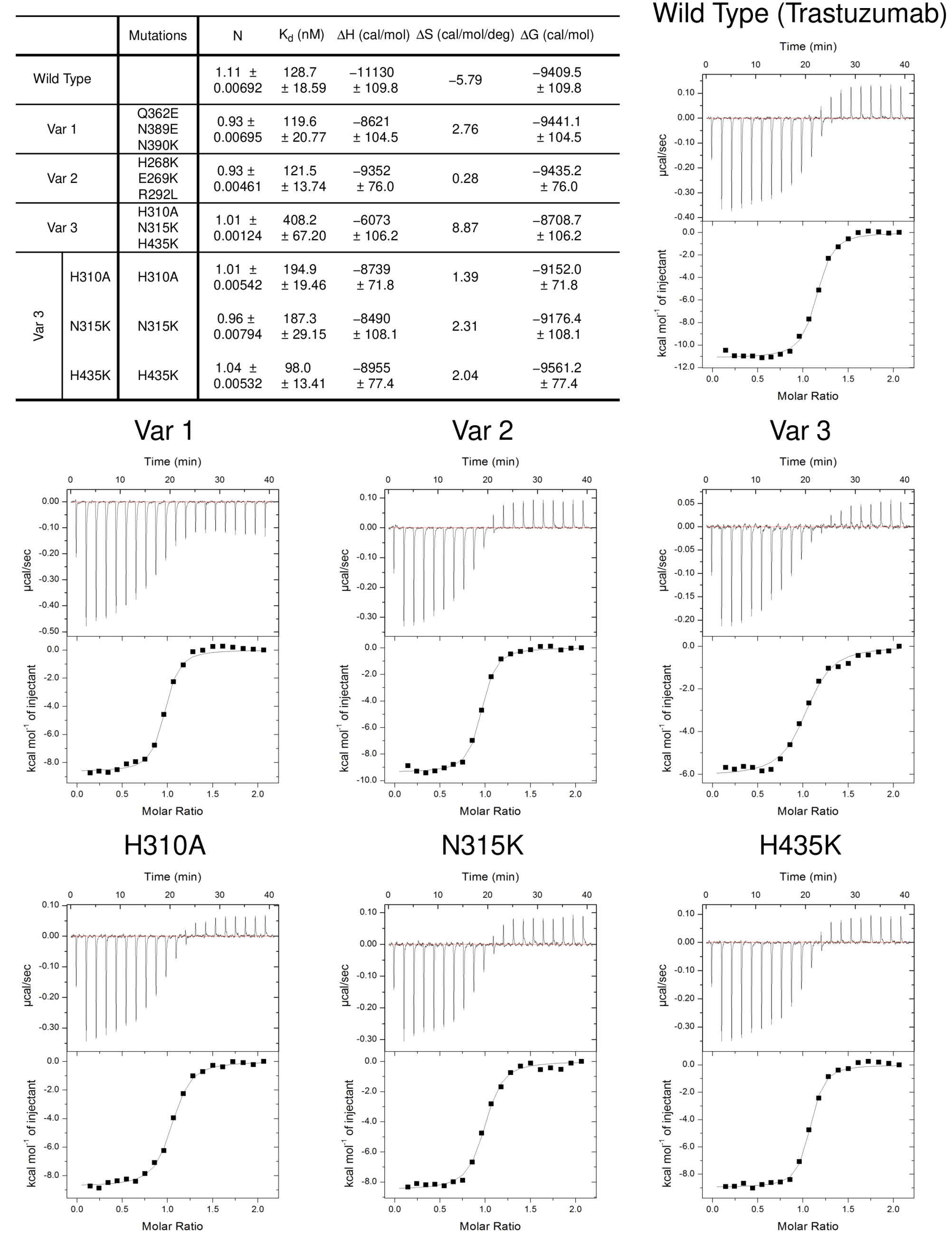

Supplement: S2 Fig — Based on the Kd values, H310 and N315 overlap epitopes. (TIF) [file pcbi.1008150.s002.tif]

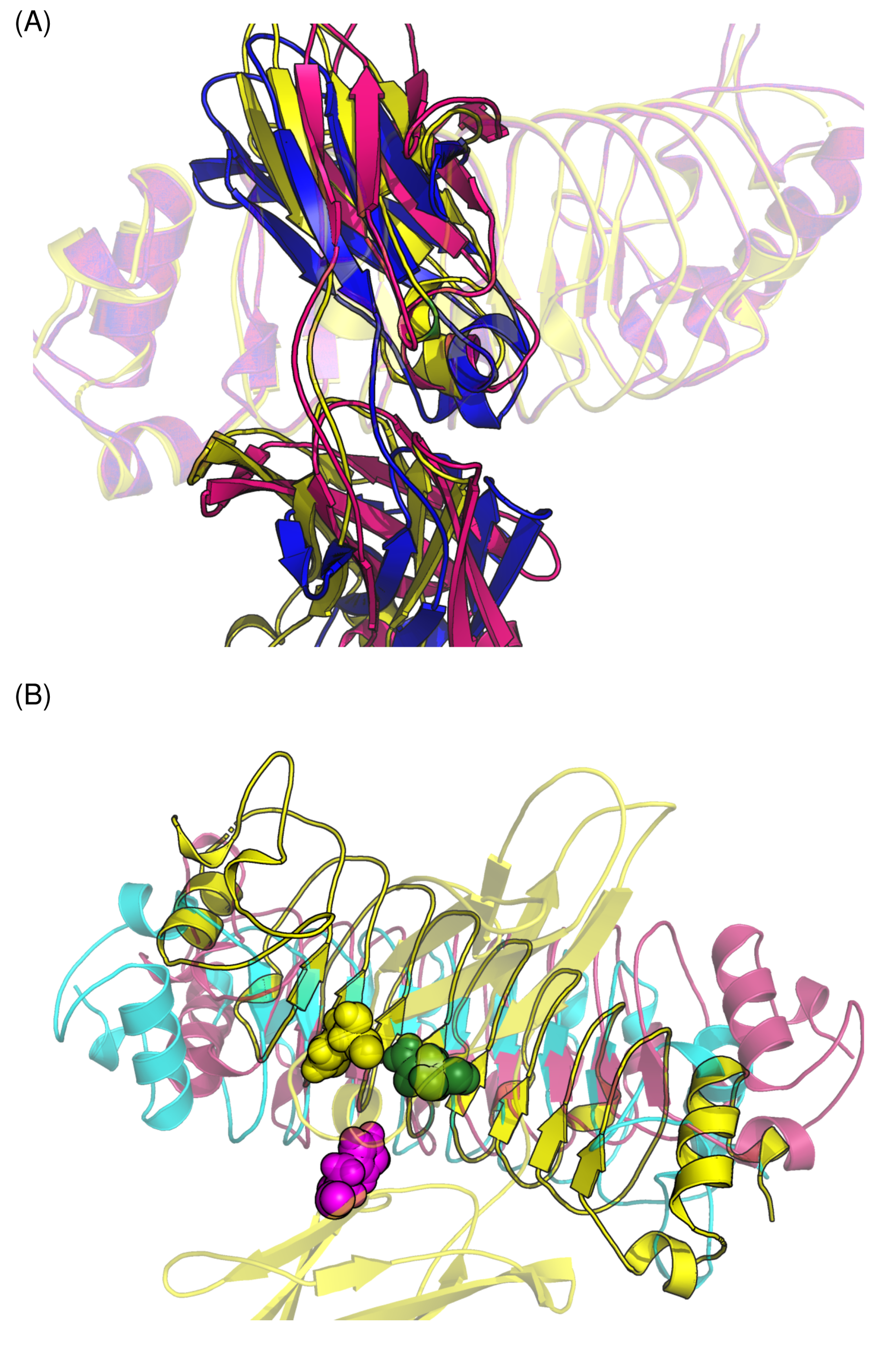

Supplement: S3 Fig — The crystal structure is in gold. (A) The full atom energy minimization step may change the overall structure, but the binding interactions are likely to be maintained. The I-RMSD value of the lowest energy model (Model 1, blue) is slightly higher than the model with the second lowest energy (Model 2, pink). However, its fnat is twice higher. (B) There are two docking models which are in contact with the three mutations in Var 3 (Model 9: cyan and Model 10: pink). While their binding interface regions are largely correct, the binding orientation of the lower energy model (pink) is completely inverted. (TIF) [file pcbi.1008150.s003.tif]

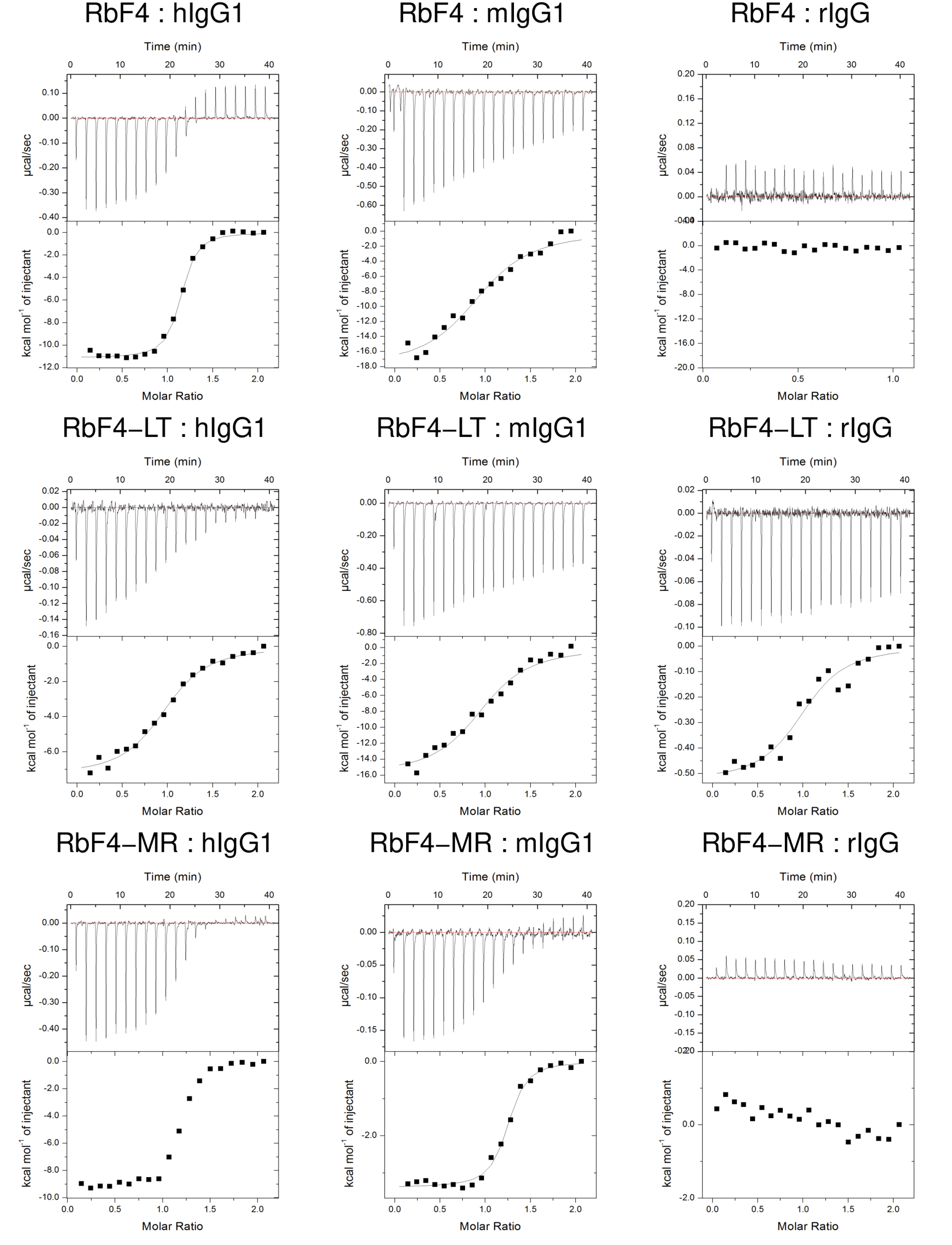

Supplement: S4 Fig — RbF4 binds strongly to hIgG1 and weakly to mIgG1. However, no binding affinity is measured for RbF4-rIgG. The truncation of the loop (RbF4-LT) enabled the variant to bind to all IgGs with similar binding affinities. RbF4-MR gains strong binding affinities for hIgG1 and mIgG1. See S2 and S3 Tables for details. (TIF) [file pcbi.1008150.s004.tif]

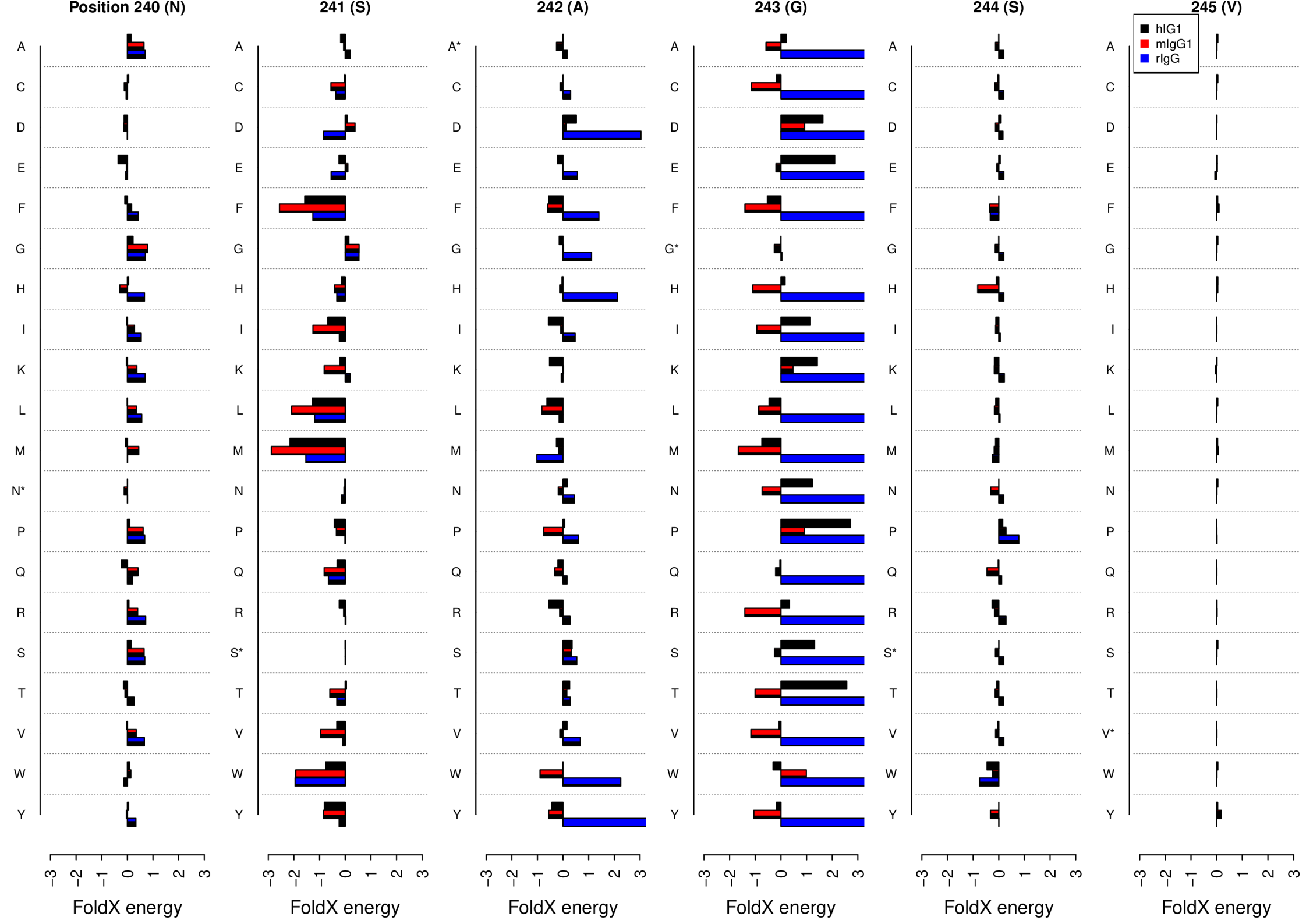

Supplement: S5 Fig — The residue scan using FoldX suggests that the inclusion of the loop may not enhance the binding affinity of RbF4 for rIgG. (TIF) [file pcbi.1008150.s005.tif]

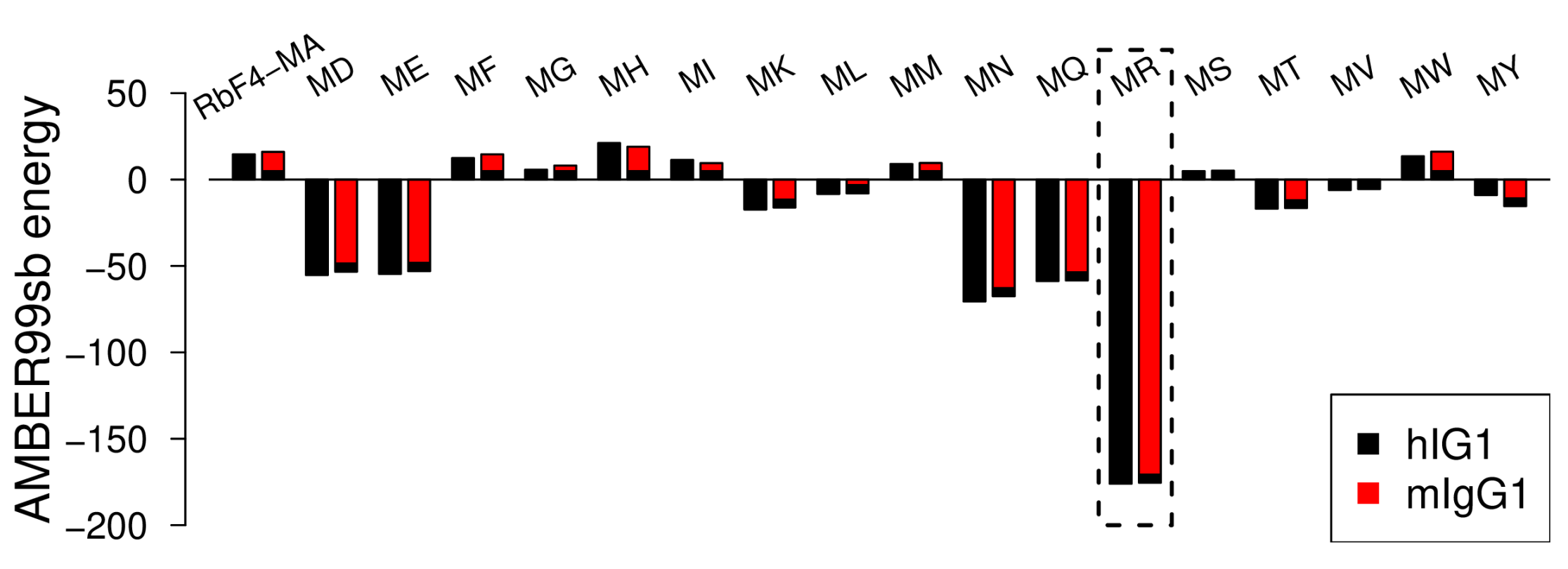

Supplement: S6 Fig — The variant with S241M and S244R mutations (RbF4-MR) is predicted to strongly bind to both hIgG1 and mIgG1. S244C and S244P were not considered. (TIF) [file pcbi.1008150.s006.tif]

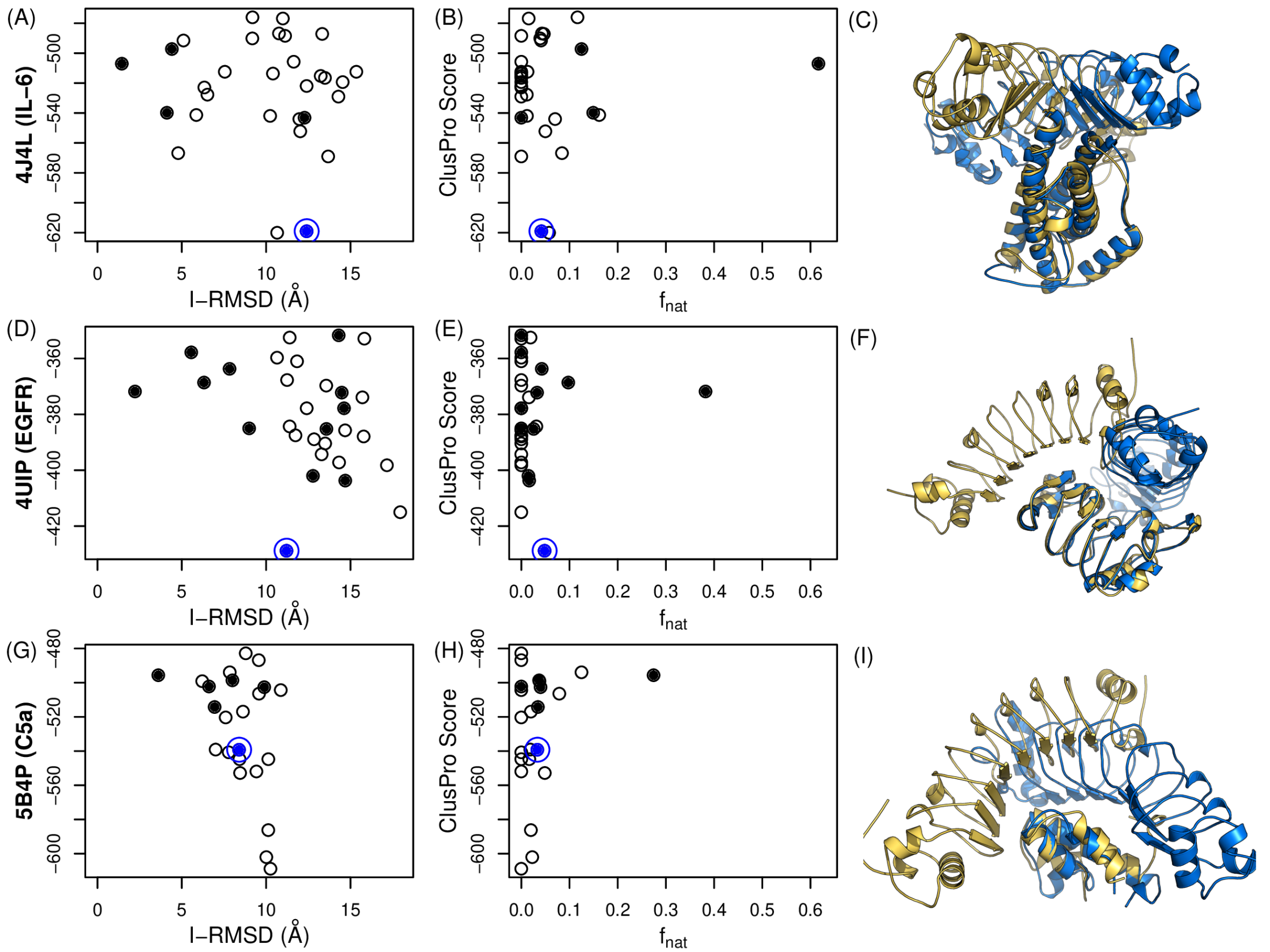

Supplement: S7 Fig — The ClusPro score was tested on the retrospective test set (A-C: IL-6, D-F: EGFR, and G-I: C5a binders). Docking models that are in contact with epitope overlapping residues (localized docking models) are in solid circle. The blue circle is the model with the lowest ClusPro score. Crystal structures are in yellow and the docking models with the lowest energies are in blue on the right hand side. Score assessment using the ClusPro score is not predictive. (TIF) [file pcbi.1008150.s007.tif]
