# Supplementary material for: Computer-guided binding mode identification and affinity improvement of an LRR protein binder without structure determination
Source: PLoS Comput Biol. 2020 Aug 31;16(8):e1008150. doi: 10.1371/journal.pcbi.1008150 (PMC7485979; doi:10.1371/journal.pcbi.1008150)
Supplement: S1 Table — (DOCX) [file pcbi.1008150.s008.docx]

**S1 Table. Data collection and refinement statistics**

|  | RbF4/hFc |
| --- | --- |
| **Data collection** |  |
| Space group | P 2_1_2_1_2_1_ |
| Cell dimensions |  |
| *a*, *b*, *c* (Å) | 59.9, 107.4, 171.4 |
| *α*, *β*, *γ* (°) | 90,90,90 |
| Resolution (Å) | 30-3.0 (3.05-3.00)* |
| *R*_merge_ (%) | 17.5(100.0) |
| *I/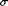I* | 15.0(1.7) |
| Completeness (%) | 100(100) |
| Redundancy | 13.2(12.6) |
|  |  |
| **Refinement** |  |
| Resolution (Å) | 30-3.0 |
| No. reflections | 22835 |
| *R*_working_ / *R*_free_ (%) | 25.7 / 33.3 |
| No. atoms |  |
| Protein | 6613 |
| Ligand/ion | 99 |
| water | 6 |
| B-factors |  |
| Protein | 56.9 |
| Ligand/ion | 76.4 |
| water | 35.2 |
| R.m.s deviations |  |
| Bond lengths (Å) | 0.010 |
| Bond angles (º) | 1.246 |

*I/σI,* mean intensity/sigma of all reflections; R_free_, Σ||F_obs_|-|F_calc_|| / Σ|F_obs_| where 5% of randomly selected data were used; R_merge_, Σ|*I*_hkl_-<*I*_hkl_>| / Σ*I*_hkl_ ; where <*I*_hkl_> is the mean intensity of all reflections equivalent to refection hkl; R_work_, Σ||F_obs_|-|F_calc_|| / Σ|F_obs_|.

*Highest resolution shell is shown in parenthesis.
