# Supplementary material for: Computer-guided binding mode identification and affinity improvement of an LRR protein binder without structure determination
Source: PLoS Comput Biol. 2020 Aug 31;16(8):e1008150. doi: 10.1371/journal.pcbi.1008150 (PMC7485979; doi:10.1371/journal.pcbi.1008150)
Supplement: S2 Table — Each black block indicates that the Fc position is in contact with the docked repebody. (DOCX) [file pcbi.1008150.s009.docx]

**S2 Table. Quality measures and contact information of RbF4 docking models.** Each black block indicates that the Fc position is in contact with the docked repebody.

| **Model** | **AMBER99sb total energy** | **I-RMSD** | **f_nat_** | **Var 1** | | | **Var 2** | | | **Var 3** | | |
| --- | --- | --- | --- | --- | --- | --- | --- | --- | --- | --- | --- | --- |
|  |  |  |  | **Q362** | **N389** | **N390** | **H268** | **E269** | **R292** | **H310** | **N315** | **H435** |
| **1** | -23025.43 | 2.886 | 0.434 |  |  |  |  |  |  |  |  |  |
| **2** | -23002.86 | 2.323 | 0.292 |  |  |  |  |  |  |  |  |  |
| **3** | -22984.81 | 14.954 | 0.044 |  |  |  |  |  |  |  |  |  |
| **4** | -22981.08 | 15.629 | 0.057 |  |  |  |  |  |  |  |  |  |
| **5** | -22978.22 | 15.264 | 0.016 |  |  |  |  |  |  |  |  |  |
| **6** | -22964.96 | 4.057 | 0.041 |  |  |  |  |  |  |  |  |  |
| **7** | -22957.28 | 6.922 | 0.000 |  |  |  |  |  |  |  |  |  |
| **8** | -22997.36 | 16.006 | 0.056 |  |  |  |  |  |  |  |  |  |
| **9** | -22990.97 | 3.901 | 0.279 |  |  |  |  |  |  |  |  |  |
| **10** | -23033.53 | 14.573 | 0.000 |  |  |  |  |  |  |  |  |  |
| **11** | -23012.27 | 14.588 | 0.046 |  |  |  |  |  |  |  |  |  |
| **12** | -23010.70 | 14.071 | 0.046 |  |  |  |  |  |  |  |  |  |
| **13** | -23010.70 | 13.929 | 0.000 |  |  |  |  |  |  |  |  |  |
| **14** | -22997.03 | 15.852 | 0.015 |  |  |  |  |  |  |  |  |  |
| **15** | -22994.80 | 12.450 | 0.000 |  |  |  |  |  |  |  |  |  |
| **16** | -22991.84 | 11.205 | 0.000 |  |  |  |  |  |  |  |  |  |
| **17** | -22989.76 | 15.473 | 0.017 |  |  |  |  |  |  |  |  |  |
| **18** | -22984.90 | 19.531 | 0.000 |  |  |  |  |  |  |  |  |  |
| **19** | -22983.64 | 12.852 | 0.000 |  |  |  |  |  |  |  |  |  |
| **20** | -22978.22 | 15.716 | 0.000 |  |  |  |  |  |  |  |  |  |
| **21** | -22976.98 | 15.060 | 0.000 |  |  |  |  |  |  |  |  |  |
| **22** | -22976.54 | 15.383 | 0.000 |  |  |  |  |  |  |  |  |  |
| **23** | -22973.94 | 8.051 | 0.000 |  |  |  |  |  |  |  |  |  |
| **24** | -22970.76 | 18.285 | 0.017 |  |  |  |  |  |  |  |  |  |
| **25** | -22965.44 | 14.074 | 0.000 |  |  |  |  |  |  |  |  |  |
| **26** | -22957.28 | 10.174 | 0.000 |  |  |  |  |  |  |  |  |  |
| **27** | -22948.56 | 12.976 | 0.000 |  |  |  |  |  |  |  |  |  |
| **28** | -22945.15 | 14.537 | 0.000 |  |  |  |  |  |  |  |  |  |
| **29** | -22936.86 | 15.529 | 0.000 |  |  |  |  |  |  |  |  |  |
