# Supplementary material for: Computer-guided binding mode identification and affinity improvement of an LRR protein binder without structure determination
Source: PLoS Comput Biol. 2020 Aug 31;16(8):e1008150. doi: 10.1371/journal.pcbi.1008150 (PMC7485979; doi:10.1371/journal.pcbi.1008150)
Supplement: S3 Table — RbF4 strongly binds to hIgG1 (weakly to mIgG1), but no binding to rIgG is observed. Removal of the loop (RbF4-LT) causes a slight reduction in the binding affinity toward hIgG1, but yields a marginal affinity improvement for mIgG1. RbF4-LT produces a significant improvement in the affinity for rIgG. RbF4 with two mutations at 241 and 244 (S241M with S244R, RbF4-MR) binds to both hIgG1 and mIgG1 with high binding affinities. (DOCX) [file pcbi.1008150.s010.docx]

**Table S3. In silico binding specificity control and affinity improvement.** RbF4 strongly binds to hIgG_1_ (weakly to mIgG_1_), but no binding to rIgG is observed. Removal of the loop (RbF4-LT) causes a slight reduction in the binding affinity toward hIgG_1_, but yields a marginal affinity improvement for mIgG_1_. RbF4-LT produces a significant improvement in the affinity for rIgG. RbF4 with two mutations at 241 and 244 (S241M with S244R, RbF4-MR) binds to both hIgG_1_ and mIgG_1_ with high binding affinities.

| **Target** | **hIgG_1_** | | | **mIgG_1_** | | | **rIgG** | | |
| --- | --- | --- | --- | --- | --- | --- | --- | --- | --- |
| **Rb** | **RbF4** | **RbF4-LT** | **RbF4-MR** | **RbF4** | **RbF4-LT** | **RbF4-MR** | **RbF4** | **RbF4-LT** | **RbF4-MR** |
| **K_d_ (nM)** | 128.70 | 598.80 | 125.79 | 1009.08 | 775.19 | 168.07 | N. D. | 1189.06 | > 6 μM |
| **N** | 1.01 ± 0.0092 | 0.984 ± 0.0190 | 1.19 ± 0.0078 | 0.964 ± 0.0356 | 0.989 ± 0.0326 | 1.21 ± 0.0099 | 7.83e4 ± 5.03e12 | 1.01 ± 0.0455 | 1.87 ±0.173 |
| **ΔH (cal/ mol)** | -8102 ±110.7 | -7335 ±198.9 | -9394 ±100.5 | -2.1e4 ±1139 | -1.593e4 ± 752.6 | -3386 ±43.58 | -325.8 ±162.7 | -529.9 ±33.4 | 2107 ±341.9 |
| **ΔS (cal/ mol/ deg)** | 2.65 | 3.9 | 0.08 | -43.6 | -25.5 | 19.6 | 36.3 | 25.3 | 30.7 |
