# Supplementary material for: Computer-guided binding mode identification and affinity improvement of an LRR protein binder without structure determination
Source: PLoS Comput Biol. 2020 Aug 31;16(8):e1008150. doi: 10.1371/journal.pcbi.1008150 (PMC7485979; doi:10.1371/journal.pcbi.1008150)
Supplement: Attachment — Submitted filename: response_final.pdf [file pcbi.1008150.s011.pdf]

RESPONSE TO REVIEWER COMMENTS
(Reviewer comments in light blue; responses in plain text)

We thank the editor and reviewers for thoroughly evaluating our manuscript and providing helpful comments. We have addressed each concern, as elaborated and clarified in the following responses.

There was a mistake in **Table 1** (PDB ID of the RbF4-Fc complex structure: 5Z7K $\rightarrow$ 6KA7). We have added one additional supplementary table (**Table S2**) and a figure (**Figure S3**) to the manuscript. The manuscript has been substantially edited for English correction, including the title of the manuscript. All the changes related to responses are highlighted in red in the tracked manuscript.

Response to Reviewer #1

My first concern is that the authors seem to make generalizations in the introduction but cite single articles which make the point. While the generalizations are correct there are better references the authors could cite. Page 4 line 64 the authors cite references 10, 11 for the current status of prediction protein:protein binding poses. The authors should cite a comparative study such as:

1. Huang S-Y. Exploring the potential of global protein–protein docking: an overview and critical assessment of current programs for automatic ab initio docking. Drug Discov Today. Elsevier Ltd; 2015;20: 969–977. doi:10.1016/j.drudis.2015.03.007
2. Hogues H, Gaudreault F, Corbeil CR, Deprez C, Sulea T, Purisima EO. ProPOSE: Direct exhaustive protein-protein docking with side chain flexibility. J Chem Theory Comput. 2018;14: 4938–4947. doi:10.1021/acs.jctc.8b00225

Page 4 line 64 the authors cite reference 12 with respect to the challenge of prediction protein:protein affinity. The authors should cite a comparative study or a more relevant review such as:

1. Sirin S, Apgar JR, Bennett EM, Keating AE. AB-Bind: Antibody binding mutational database for computational affinity predictions. Protein Sci. 2016;25: 393–409. doi:10.1002/pro.2829
2. Kastritis PL, Moal IH, Hwang H, Weng Z, Bates PA, Bonvin AMJJ, et al. A structure-based benchmark for protein-protein binding affinity. Protein Sci. 2011;20: 482–491. doi:10.1002/pro.580
3. Siebenmorgen T, Zacharias M. Computational prediction of protein–protein binding affinities. Wiley Interdiscip Rev Comput Mol Sci. 2019; 1–18. doi:10.1002/wcms.1448

As the reviewer commented, we have added the references suggested by the reviewer to the revised manuscript (**page 4 line 66**, **references 10, 12, 15, 16 and 17**).

While not a concern, on Page 6 line 113-115 the authors state that they "… assumed that the major driving force of RbF4 for target binding should be no different from antibodies or high affinity protein binders (Antibody Mode)". While the authors clearly show the benefit of using the Antibody mode scoring function in ClusPro in table 1, I would like the authors to expand on this statement and explain which features they feel are similar between their binding event and that of antibody CDR interacting with an antigen. In the reference cited by the authors (reference 28) the ClusPro authors state that antibody recognition is typically flatter and less hydrophobic than enzyme pockets, is this similar for your binding event. Additional discussion and insight would be beneficial to the reader.

The original paper for the antibody mode (Brenke et al. (2012)) showed that the DARS (Decoys As the Reference State) energy term, which was proven to be useful for general protein-protein docking simulations, worsens the prediction accuracy of antibody-antigen pairs. The authors argued that it may be due to the asymmetric nature of antibody-antigen interactions, which should also be true for other protein binders. Considering the structural features of LRR proteins, their antigen recognitions seem to be flatter as described in reference 28, but the degree of hydrophobicity compared with enzyme pockets is unclear. We included this discussion in the revised manuscript (**page 6 line 124 and page 11 line 219**).

Page 7 line 136 does the author mean AMBER99sb forcefield energy? Is this the total energy including the internal energy (bond, angle, torsions)?

It is the total energy including all other terms. We have clarified which energy we used to rank docking models in the revised manuscript (**page 7 line 145, page 10 line 196, and page 15, line 321**).

On Page 9 Line 172-173 the authors state the following "The results indicate that the predicted binding mode is sufficiently accurate for further engineering of the binding specificity." While the authors results are great, we should be careful about people misreading this sentence. The authors should clearly state that the predicted binding mode is the result of not only using a docking tool and scoring function but results were filtered using experimental information.

Based on the reviewer's comments, we have clarified that the binding mode identification was achieved in combination with computational molecular docking and experimental information in the revised manuscript (**page 9 line 188**).

On page 9 line 179 the authors mention selection of LRR designs using AMBER forcefield energy yet this is not described in the experimental protocol on page 14. Did you use AMBER99sb again? Did you use total energy or MM-GB/SA? Clarification is needed.

As the reviewer suggested, we have clarified that the force field used for the improvement of binding affinity was AMBER99sb as consistently used in the binding mode identification. We have described that the total energy was used to rank model structures in the method section of the revised manuscript (**page 10**, **line 196 and page 15 line 322**).

On page 9 line 180 the authors mention a FoldX scan, but no mention of this in the computational methods section. Could the authors please include this. Also, to follow up on the above point did the authors solely use FoldX to create the mutants then use AMBER to score them or did they use the FoldX binding affinity scoring function? Again, further clarification is needed.

In our previous study (Choi *et al.* (2018)), we found that FoldX is extremely good at discriminating binding disruptive mutations (but not vice versa). In order to fast remove mutations not helpful to improve binding affinity, we employed FoldX. We have clarified why FoldX was used (**page 10 line 197**) and added the description of FoldX commands used in our study (**page 15 line 321**).

On Page 14 line 280-283 the authors state “From the two studies of the binding mode prediction and affinity improvement, the force field energy prediction becomes informative only if actual binding is known, i.e. supportive data from experiments are critical in practice.” I believe the authors are referring to their work here and therefore they should explicitly state that. Also, the statement is partially incorrect, since the authors clearly show that the forcefield energy can discriminate docking poses, but not how it correlates with binding affinity. This should be removed.

We agree with the reviewer that our description is contradictory to our work. We have removed it from the manuscript (**page 15 line 308**).

Response to Reviewer #2

Major comments:

1) The authors need to make clear in the introduction to the study the relationship of the work carried out in this manuscript to the previously published protocol from ref [10]. It is not immediately clear to the reader that the approach described in this manuscript is not wholly original, and I found that to be somewhat misleading. Placing the work in the broader context of the field does not detract from it.

As the reviewer pointed out, this work could be considered as an extended application of our previous work on antibody epitope localization (Now, ref. #11). Considering the structural feature of the protein binder used and the final goal of the present work, we thought that this work could be independent of the previous one. We now have clarified that this work is based on the previously published method and what is the main difference in the Introduction section (**page 5 line 80**).

2) How did the authors confirm that the measured reduction in binding affinity was not caused by changes in the stability or structure of Var3, H75A or N80K, but rather resulted from specific disruption of the Ab-Ag binding interface?

We did not experimentally confirm that the measured reduction in binding affinity was not caused by changes in the stability or structure of the mutants. But, the computational method takes structural stability into account while calculating binding disruption. As shown in **Fig. 1B**, the decreases in binding affinities of H75A (now H310A, see our response in the below comment) and N80K (N315K) are rather marginal and the disruption level of Var 3 seems like the addition of the two single mutations. Affinity decreases would be more dramatic than those shown in the figure if the mutations caused significant structural instability. Thus, we think that reduced affinity of the mutants is mainly due to the specific disruption of the binding interface.

3) What positions were contained in Var 1 and Var 2? Please provide position numbering that is concordant the the PDB structure 3AVE referred to in the text.

The mutations for Var 1 and 2 were listed in **Figure S2**. We have specified the mutations in the main manuscript as well (**page 7, line 131**). As the reviewer pointed out, we have changed the position numbering in concord with 3AVE in the revised manuscript.

4) The decision to evaluate each of the three mutations in Var 3 as single mutants appears to deviate from the approach proposed in ref 10, requiring additional experimental work. The authors comment:
Lines 125-126 'Although the hFc interface region in contact with RbF4 was roughly identified through the triplet, it is probable that not all of them are involved in the binding.'

What is the effect on the subsequent analysis if this step to interrogate each of the three mutations in Var 3 is not taken? What do the authors recommend to future users of this technology as the standard approach?

It was observed that not all three mutations were involved in antibody-antigen binding (Hua *et al.* (2017)). There is a trade-off between efficiency and precision of epitope localization, and three mutations per design was the optimal number. To clarify this point, we have rephrased the sentence with the reference (**page 7, line 135**).

Assuming that we did not test each single mutation, one may presume either all of the three mutations in Var 3 are involved in binding or at least one of the three mutations does. In the former case, there are only two docking models (Model 8 and 9 in **Table S2**). A newly added figure (**Figure S3B**) depicts the binding orientations of the two models (Model 8: cyan, Model 9: pink and the crystal structure: yellow). While the binding interface is largely similar, the binding orientation of the model with a lower energy value (pink) is completely reversed. In the latter case, the results remain the same as reported in the manuscript. However, it is still recommended that additional experimental verification seems extremely beneficial, and one should not assume that all three positions are in contact with the binding partner. We have added a discussion on this matter to the revised manuscript (**page 8**, **line 160**).

5) Figure 2B and C show that of the docking models in contact with the epitope overlapping residues, the model with the lowest energy has close to the lowest I-RMSD and the highest f_nat. How different is the binding mode of the model with higher energy and lower I-RMSD?

The full atom energy minimization process changes backbone structures as well. The structure of a repebody remains largely the same, whereas the Fc region does not. The major structural difference between the two models comes from Fc (more precisely, the loop linking CH2 and CH3). For clarification, we have added a new figure (**Figure S3A**) to show where the structural difference comes from. Though there is a larger structural distortion observed in Model 1 (see **Table S2**) than Model 2, atomic interactions (reflected in f_{nat}) are better conserved in Model 1. We have described this point in the revised manuscript (**page 8**, **line 151**).

6) Similarly, how different were the models with lower energy in Figs 2B and C that did not fulfill the conditions of being in contact with H75 and N80 but not with H200 or the six other positions in Var 1 and 2? Please provide a table in the supplement with details of the positions in contact in each model, and the force field energy and structural parameters (I-RMSD, f_nat) for each of the 29 models.

The model with the lowest energy is in contact with Var 2. Please refer to our answer for comment 4. As the reviewer suggested, we have provided full information of each docking model in the supplementary materials (**Table S2**).

Minor comments:

1. While overall the writing and presentation is fully adequate and relatively easy to understand, the paper would nonetheless benefit greatly from careful reading to fix a large number of awkward and/or unclear statements and phrases. For example:

(i) Lines 111-113 are quite unclear, in particular the phrase 'assigning attractions at the concave residues of LRRV modules from 2 to 4, as known during the phage display selection' does not make sense.

We have thoroughly reviewed the manuscript and corrected awkward sentences and phrases, including the one the reviewer mentioned.

2. What is shown in red in Figure 1d? I'm guessing it is the repebody loop?

We have modified the legend of **Figure 1** and stated that the repebody loop is highlighted in red stick.
